# Role of *Aedes aegypti* (Linnaeus) and *Aedes albopictus* (Skuse) in local dengue epidemics in Taiwan

**DOI:** 10.1186/s12879-016-2002-4

**Published:** 2016-11-09

**Authors:** Pui-Jen Tsai, Hwa-Jen Teng

**Affiliations:** 1 Center for General Education, Aletheia University, New Taipei City, 25103 Taiwan, ROC; 2 Center for Diagnostics and Vaccine Development, Centers for Disease Control, Taipei, Taiwan, ROC

**Keywords:** Dengue fever, *Aedes* mosquitoes, Breteau index, Global bivariate Moran’s I, Geographically weighted regression

## Abstract

**Background:**

*Aedes* mosquitoes in Taiwan mainly comprise *Aedes albopictus* and *Ae. aegypti*. However, the species contributing to autochthonous dengue spread and the extent at which it occurs remain unclear. Thus, in this study, we spatially analyzed real data to determine spatial features related to local dengue incidence and mosquito density, particularly that of *Ae. albopictus* and *Ae. aegypti*.

**Methods:**

We used bivariate Moran’s I statistic and geographically weighted regression (GWR) spatial methods to analyze the globally spatial dependence and locally regressed relationship between (1) imported dengue incidences and Breteau indices (BIs) of *Ae. albopictus*, (2) imported dengue incidences and BI of *Ae. aegypti*, (3) autochthonous dengue incidences and BI of *Ae. albopictus*, (4) autochthonous dengue incidences and BI of *Ae. aegypti*, (5) all dengue incidences and BI of *Ae. albopictus*, (6) all dengue incidences and BI of *Ae. aegypti*, (7) BI of *Ae. albopictus* and human population density, and (8) BI of *Ae. aegypti* and human population density in 348 townships in Taiwan.

**Results:**

In the GWR models, regression coefficients of spatially regressed relationships between the incidence of autochthonous dengue and vector density of *Ae. aegypti* were significant and positive in most townships in Taiwan. However, *Ae. albopictus* had significant but negative regression coefficients in clusters of dengue epidemics. In the global bivariate Moran’s index, spatial dependence between the incidence of autochthonous dengue and vector density of *Ae. aegypti* was significant and exhibited positive correlation in Taiwan (bivariate Moran’s index = 0.51). However, *Ae. albopictus* exhibited positively significant but low correlation (bivariate Moran’s index = 0.06). Similar results were observed in the two spatial methods between all dengue incidences and *Aedes* mosquitoes (*Ae. aegypti* and *Ae. albopictus*).

The regression coefficients of spatially regressed relationships between imported dengue cases and *Aedes* mosquitoes (*Ae. aegypti* and *Ae. albopictus*) were significant in 348 townships in Taiwan. The results indicated that local *Aedes* mosquitoes do not contribute to the dengue incidence of imported cases.

The density of *Ae. aegypti* positively correlated with the density of human population. By contrast, the density of *Ae. albopictus* negatively correlated with the density of human population in the areas of southern Taiwan. The results indicated that *Ae. aegypti* has more opportunities for human–mosquito contact in dengue endemic areas in southern Taiwan.

**Conclusions:**

*Ae. aegypti*, but not *Ae. albopictus*, and human population density in southern Taiwan are closely associated with an increased risk of autochthonous dengue incidence.

## Background

Dengue fever (DF) is a serious mosquito-borne viral infectious disease, mostly distributed in tropical regions [1]. DF epidemics were first recognized nearly simultaneously in Asia, Africa, and North America in the 1780s [2]. Later, DF epidemics were mostly reported in Southeast Asia, particularly after the end of World War II [3]. DF epidemics became more frequent and extended to Latin America in the early 1980s [4]. Dengue virus (DENV), with a nearly ubiquitous distribution in tropical regions, was more recently introduced in Europe [5]. In DENV circulation areas, 3.97 billion people are estimated to have a risk of dengue infections [6]. Moreover, approximately 50 million clinical cases annually occur in more than 100 countries worldwide [7]. Of these, 2.5 % of dengue cases become more severe and progress into dengue hemorrhagic fever (DHF) and/or dengue shock syndrome (DSS). These severe forms of the disease are responsible for high morbidity and fatal outcomes. Approximately 5 % of patients with DHF or DSS are estimated to die, predominantly children younger than 15 years old [8]. However, in recent years, severe dengue cases have tended to increase among adults in some areas [9]. DENV is the etiological agent of DF, DHF, and DSS. A sylvatic cycle that serves as an enzootic cycle involving monkeys and jungle mosquitoes may be present [10]. A study reported that the currently circulating DENV-1 may have resulted from a spillover of ancestral sylvatic strains [11]. DENV is one of the 70 members of the genus Flavivirus of the family Flaviviridae, which consists of four closely related but genetically distinct antigenic serotypes, namely DENV-1, -2, -3, and -4, originally classified based on their serological characteristics [12]. Each DENV serotype elicits neutralizing antibodies in infected humans, resulting in lifelong immunity against the corresponding virus serotype [3]. DENV can infect various tissues in mosquito vectors, particularly the midgut and salivary glands [13].

The mosquitoes *Aedes aegypti* and *Ae. albopictus* [14] are vectors of several globally critical arboviruses including DENV [15], yellow fever virus [16], chikungunya virus [17], and Zika viruses [18]. *Ae. aegypti* originated in Africa, where its ancestral form was a zoophilic tree-hole mosquito named *Ae. aegypti formosus* [19]. The domestic form of *Ae. aegypti* is genetically distinct with discrete geographic niches [20]. It has been hypothesized that harsh conditions coupled with the onset of the slave trade resulted in the introduction of *Ae. aegypti* into the new world from Africa, from where it subsequently spread into tropical and subtropical regions of the world [19]. *Ae. albopictus*, originally a zoophilic forest species from Asia [21], spread to islands in the Indian and Pacific Oceans [22]. During the 1980s, *Ae. albopictus* rapidly expanded into Europe, the United States, Brazil, and Central Africa [23–25].

Taiwan is geographically located in a region that spans both tropical and subtropical climates (22–25°N and 120–122°E). Although both *Ae. aegypti* and *Ae. albopictus* are prevalent in Taiwan, they have different distributions [26, 27]. *Ae. albopictus* is extensively distributed at elevations of less than 1500 m throughout the island, whereas *Ae. aegypti* appears only in the south below the Tropic of Cancer (i.e., 23.5°N). The Taiwan Centers for Disease Control (CDC) reported similar results based on surveys of the dengue vector *Ae. aegypti* conducted in Taiwan during 1988–1996 and 2003–2004 [27]. However, the reason behind the absence of *Ae. aegypti* in northern Taiwan remains unclear. This absence may be attributable to the sensitivity of *Ae. aegypti* to lower winter temperatures in the north, resulting in their limited distribution in Taiwan [28]. The same observations were reported in the temperate and subtropical regions of Argentina (e.g., the city of Resistencia, Chaco). The mortality rate of *Ae. aegypti* eggs due to lower winter temperatures was 48.6 % [29]. DF is a travel-related disease in Taiwan because travelers can carry DENV from endemic areas into the island [30–33]. After the transport of this virus into the island, it is passed on to *Aedes* mosquitoes, which can cause an outbreak of DF. Historical epidemics of dengue in Taiwan were documented in 1902, 1915, and 1922 in Penghu Islet (i.e., Penghu County); 1924 and 1927 in southern Taiwan; 1931 in Tainan; and 1942–1943, island-wide [34]. A reason for these epidemics was the high prevalence of water storage tanks among households during wartime and the entry of dengue patients from epidemic or endemic countries into Southeast Asia. The virus was silent for almost 37 years until 1981, when the DEN-2 DF epidemic occurred on the islet of Hsiao-LiuChiu (i.e., Liouciou Township), which is located off southern Taiwan [35]. However, it did not cause an epidemic in mainland Taiwan. A DENV-1 epidemic occurred in 1987–1988 in southern Taiwan. In 1987–2002, the epidemic patterns of dengue in Taiwan cycled with small-scale outbreaks occurring nearly every 3 years and large-scale epidemics occurring nearly every 10 years [36]. However, since 2002, the Taiwan CDC have recorded nine outbreaks with more than 1000 cases of DF and/or DHF/DSS in 2002, 2006, 2007, 2009, 2010, 2011, 2012, 2014, and 2015, respectively [37]. With a few exceptions, these outbreaks occurred in the south of Taiwan where *Ae. aegypti* is prevalent and coexists with *Ae. albopictus*. The relationship between vectors and autochthonous dengue incidence in terms of geographical correlation has not yet been quantitatively assessed. Therefore, the role of *Aedes* mosquitoes (i.e., *Ae. aegypti* and *Ae. albopictus*) in local dengue epidemics has largely been neglected in spatial research. Therefore, this study investigated the role of *Aedes* mosquitoes in the occurrence of autochthonous dengue and the effects of human population density on the distribution of *Aedes* mosquitoes in Taiwan.

## Methods

### Surveillance of *Aedes* mosquitoes in the main island of Taiwan

**Fig. 1:**
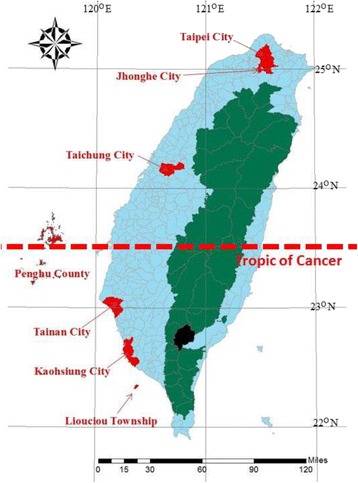
Map of townships in the study area throughout the main island of Taiwan and excluded studied areas of Penghu County and Liouciou Township. A total of 348 studied areas consisted of 294 townships on plain regions (blue and red boxes) and 54 aboriginal townships in mountainous regions (green box), except Wutai Township (black box)

The mosquito data set used in this study was a part of national routine entomological surveillance of dengue and the original data collected in a 3-year project of Taiwan CDC through 2009-2011 [27]. In brief, a sample of 100 mosquito larvae was collected from each of the 7,019 subtownships (i.e., villages or communities; the smallest administrative units in this study) in the main island of Taiwan between 2009 and 2011. In the villages, the mosquito larvae sampling frequency ranged from 1 to 130 over the 3 years, and the mean sampling frequency was 23. All samples were sent to the CDC for laboratory analysis. All mosquito classification data from the 7,019 villages were categorized into 365 townships. In this study, we focused on the main island of Taiwan for detecting spatial contiguity and pattern consistency. Thus, we excluded townships belonging to outlying islands and investigated a total of 348 townships (Wutai Township, which is located in a remote mountainous area, was the only township on the main island of Taiwan that was not investigated; Fig. 1). The incidence rates of *Ae. albopictus* in each township were calculated using the number of confirmed *Ae. albopictus* larvae as a numerator and a total of confirmed *Aedes* larvae as a denominator. The incidence rates of *Ae. aegypti* were calculated using the same formula.

The Breteau index (BI) is defined as the number of positive containers per 100 houses inspected [38]. We used averaged BI values to represent the dengue vector density in a given area. BI data for the period between 2009 and 2011 were obtained from the CDC. A total of 115,690 BI records for the villages were archived into 348 townships and were used for calculating the average BI. For each township, the BI of *Ae. albopictus* was calculated by multiplying the incidence rate of *Ae. albopictus* with the average BI, and the BI of *Ae. aegypti* was calculated using the same formula.

### Data collection for confirmed dengue cases

The data of confirmed imported and autochthonous DF cases were obtained from the Taiwan National Infectious Disease Statistics System of the CDC [37]. Because DF is a notifiable disease, blood samples from patients with suspected DF symptoms are collected and sent to the CDC for laboratory confirmation. Patients who had any of the following condition will be considered to have DENV infection: (i) positive virus isolation; (ii) positive result of real-time polymerase chain reaction; (iii) positive result of higher titers of dengue-specific IgM and IgG antibody in which cross-reaction to Japanese encephalitis had been excluded; or (iv) positive seroconversion or 4-fold rise in dengue-specific IgM or IgG antibody in the convalescent phase and positive result in the NS1 antigen test [32, 39].

Data were obtained for the period between 2009 and 2011. According to the definition of the CDC, epidemiological questions regarding the travel history, incubation period, and first day of illness of patients confirmed as having DF were used to identify the possible origin of dengue infection. Laboratory-confirmed dengue cases with a travel history to endemic countries within 14 days before the date of dengue onset were defined as “dengue cases imported to Taiwan” [39]. The remaining confirmed dengue cases were defined as autochthonous cases. Ethical approval for this study was not required because we used public domain data. We evaluated 348 townships on the main island of Taiwan. The demographic data used in this study were obtained from the Ministry of the Interior [40]. Between 2009 and 2011, the incidence rate of imported and autochthonous dengue cases and the average ratio of all confirmed dengue cases were calculated for each township [37]. The incidence rate was calculated as the number of confirmed dengue cases divided by the total human population in that township.

### Global Moran’s I Statistic

Global Moran’s spatial autocorrelation was used to assess the correlation among neighboring observations and to identify patterns and levels of spatial clustering in neighboring districts [41]. Moran's statistic [42], similar to the Pearson correlation coefficient, was calculated using the following formula:

1 $$ I=\frac{N}{S_O}{\displaystyle {\sum}_i{\displaystyle {\sum}_j{w}_{ij}\frac{\left({x}_i-\overline{x}\right)\left({x}_j-\overline{x}\right)}{{\displaystyle {\sum}_i{\left({x}_i-\overline{x}\right)}^2}}}} $$

where *N* is the number of districts, *w*_*ij*_ is the element in the spatial weight matrix corresponding to the observation pair *i* and *j,* and x_*i*_ and *x*_*j*_ observations for the areas *i* and *j, respectively,* with the mean $\overline{x}$, and

2 $$ {S}_O={\displaystyle {\sum}_i{\displaystyle {\sum}_j{w}_{ij}}}. $$

Because weights were row standardized (∑*w*_*ij*_ = 1), the first step in the spatial autocorrelation analysis was to construct a spatial weight matrix that contained information on the neighborhood structure for each location. Adjacency was defined as immediately neighboring administrative districts, including the district itself. Non-neighboring administrative districts were assigned a weight of zero. Spatial contiguity for polygons was defined as the property of sharing a common boundary or vertex. Contiguity analysis is a crucial method for assessing unusual features in connectivity distribution [43]. Queen’s contiguity can be used to compensate for spatial contiguity by incorporating both the Rook and Bishop relationships into a single measure [44]. The administrative districts considered in this study were highly irregular in both shape and size. The first-order queen polygon contiguity method was the most appropriate for quantifying the spatial weights matrix for the analysis of connectivity. On the basis of this approach, spatial weight or connectivity matrices were determined and used in conjunction with the global Moran’s calculations described further.

Moran’s I values may range from −1 (dispersed) to +1 (clustered). A Moran's I value of 0 suggests complete spatial randomness. A random permutation procedure recalculates a statistic many times by reshuffling data values among map units to generate a reference distribution. The obtained calculated statistic, based on the observed spatial pattern, is then compared to the reference distribution, and a pseudo significance level (pseudo *p* value) is computed. To verify that the Moran's I value significantly differed from the expected value, we used a Monte Carlo randomization test with 9,999 permutations to obtain significant values. The data values were reassigned among the N locations, providing a randomized distribution against which the observed value could be judged. If the observed I value was within the tails of this distribution, a significant spatial autocorrelation was present in the data and a pseudo *p* value was smaller than 0.05; thus, the assumption of independence among the observations could be rejected [45].

### Global bivariate Moran’s I statistic

The global bivariate Moran’s I statistic quantifies the spatial dependency between the two variables X_l_ and X_k_ in a same location *i* [46]. This yields a counterpart of a univariate Moran-like spatial autocorrelation, defined as follows:

3 $$ {\displaystyle {I}_{kl}}=\frac{Z_kW{Z}_l}{n} $$

Where *n* is the number of observations, ${z}_k=\left[{x}_k-{\overline{x}}_k\right]/{\sigma}_k$ and ${z}_l=\left[{x}_l-{\overline{x}}_l\right]/{\sigma}_l$, which have been standardized such that the mean is zero and standard deviation equals one. *W* is the row-standardized spatial weight matrix. The weight matrix defines the neighbor set for each observation with non-zero elements for neighbors and zero for the others. The significance of this bivariate spatial correlation can be typically assessed using a randomization (or permutation) approach.

In this case, observed values for one of the variables are randomly reallocated to locations (centroids of townships), and the statistic is recomputed for each such random pattern. The resulting empirical reference distribution can aid in evaluating how extreme the observed statistic is relative to its distribution under spatial randomness to produce a Moran’s I scatter plot. The Moran’s I scatter plot visualizes a spatial autocorrelation statistic as the slope of the regression line with the spatial lag (a weighted average of the value of a variable in the neighboring locations) on the vertical axis and the original variable on the horizontal axis [46].

The same analysis is true for the bivariate Moran’s I scatter plot. The slope of the linear regression through this scatter plot equals the statistic in equ. (3), yielding an interpretation of the spatial lag because inverse distance neighboring values will be used for the spatial weight matrix.

Because the Z variables are standardized, the sum of squares used in the denominator of equ. (3) is constant and is equivalent to *n*, irrespective of whether Z_k_ or Z_l_ is used. Therefore, the focus is on the linear association of a variable Z_k_ at a location i (Z_ki_) with the corresponding spatial lag of the other variable [W_zl_]_i_. This concept was derived from bivariate spatial correlation and therefore centers on the extent to which values of the variable Z_k_ observed at a given location k exhibit a systematic association with another variable Z_l_ observed at the neighboring location *i* [47].

Global bivariate Moran’s I values may range from −1 to +1. A Moran’s I value of −1, 0, and 1 indicates perfect negative spatial dependence between variables, no correlation between variables, and perfect positive spatial dependence between variables, respectively. A random permutation procedure recalculates a statistic numerous times by reshuffling data values among map units to generate a reference distribution. The obtained calculated statistic based on the observed spatial pattern was then compared to this reference distribution, and a pseudo significance level (pseudo *p* value) was computed. To verify that the Moran's I value significantly differed from the expected value, we applied a Monte Carlo randomization test with 9,999 permutations to obtain significant values. Data values were reassigned among the N locations, providing a randomized distribution against which the observed value may be judged. If the observed Moran's I value was within the tails of this distribution, significant spatial dependence was present in the data, and if there the pseudo *p* value was less than 0.05, then the assumption of independence among the observations could be rejected. Global Moran’s I statistic and Global bivariate Moran’s I statistic were calculated using GeoDa 1.4.6 [48].

### Geographically weighted regression

Geographically weighted regression (GWR) is a type of a local statistic that can produce a set of local parameter estimates demonstrating the variation of a relationship over space and then enable examination of the spatial pattern of the local estimates to gain some understanding of possible hidden causes for the pattern. GWR provides a local model of the variable or process by fitting a regression equation to every feature in the dataset. GWR constructs these separate equations by incorporating the response and explanatory variables of features falling within the bandwidth of each target feature. The shape and size of the bandwidth is dependent on the user input for the kernel type, bandwidth method, distance, and number of neighbor parameters [49].

GWR [49, 50] extends the traditional regression framework (such as the ordinary least squares model; OLS) by enabling local, rather than global, parameters to be estimated so that the model is rewritten as follows:

4 $$ {y}_i={\beta}_o\left({u}_i,{v}_i\right)+{\displaystyle \sum_j{\beta}_j\left({u}_i,{v}_i\right)}{x}_{ij}+{\varepsilon}_i $$

where y_i_ is the response variable, u_i_ and v_i_ are the coordinates for each location *i*, β_0_ (u_i_, v_i_) is the intercept for location *i*, β_j_ (u_i_, v_i_) is the continuous function β_j_ (*u*, *v*) at location *i*, x_ij_ is the ith observation of attribute x at location *j*, and ε_i_ is the error term for location *i*.

The weight assigned to each observation is based on a distance-decay function centered on observation *i*.

The estimator for the GWR model is similar to the weighted least squares global model, except that the weights are conditioned on location *u* relative to the observations in the dataset and thus change for each location. The estimator is expressed as follows:

5 $$ \widehat{\beta}(u)={\left({X}^TW(u)X\right)}^{-1}{X}^TW(u)y $$

*W*(*u*) is a square matrix of weights relative to the position *u*. A particular location can be indexed (u_j_,v_j_) in the study area. *XTW*(*u*)*X* is the geographically weighted variance-covariance matrix, and *y* is the vector of the response variable value.

The *W*(*u*) matrix contains the geographical weights in its leading diagonal and zero in its off-diagonal elements.

6 $$ \left[\begin{array}{cccc}\hfill {w}_1(u)\hfill & \hfill 0\hfill & \hfill 0\hfill & \hfill 0\hfill \\ {}\hfill 0\hfill & \hfill {w}_2(u)\hfill & \hfill 0\hfill & \hfill 0\hfill \\ {}\hfill 0\hfill & \hfill 0\hfill & \hfill \cdots \hfill & \hfill 0\hfill \\ {}\hfill 0\hfill & \hfill 0\hfill & \hfill 0\hfill & \hfill {w}_n(u)\hfill \end{array}\right] $$

The distance-decay function, which may take various forms, is modified by a bandwidth setting at a distance at which the weight rapidly approaches zero. In the area in which the present study was conducted, the sample points raised from the polygon centroids were not regularly placed, but were clustered. A convenient way of implementing the adaptive bandwidth specification is to select a kernel that allows the same number of sample points for estimations.

The weight can be calculated using the fixed spatial kernel and the value set for any observation with a distance exceeding the bandwidth to zero. The bi-square function can be expressed as follows:

7 $$ {w}_{ij}={\left(1-{\left(\raisebox{1ex}{${d}_{ij}$}\!\left/ \!\raisebox{-1ex}{$h$}\right.\right)}^2\right)}^2 $$

where w_ij_ is a continuous function of d_ij_, d_ij_ is the distance from the location *i* to *j*, w_ij_ is zero when d_ij_ > *h* (*h* represents a quantity known as the bandwidth). This is a near-Gaussian function with the useful property of the weight being zero at a finite distance.

When the values for a particular explanatory variable cluster spatially, problems with local multicollinearity likely occur. The condition number in the output feature class indicates when results are unstable due to local multicollinearity. In general, results of features having a condition number of >30 should be interpreted with caution. Problems with local collinearity can prevent both the Akaike information criterion (AIC) and cross validation (CV) bandwidth methods from resolving an optimal distance or number of neighbors.

The bandwidth was chosen by minimizing the AIC score, calculated as follows:

8 $$ AI{C}_c=2nlo{g}_e\left(\widehat{\sigma}\right)+{nlog}_e\left(2\pi \right)+n\left\{\frac{\mathrm{n}+\mathrm{t}\mathrm{r}\left(\mathrm{S}\right)}{\mathrm{n}\hbox{-} 2\hbox{-} \mathrm{yr}\left(\mathrm{S}\right)}\right\} $$

where *n* is the sample size, $\widehat{\sigma}$ is the estimated standard deviation of the error, and tr(S) is the trace of the hat matrix. The AIC method has the advantage of accounting for variation in the degrees of freedom among models centered on different observations. The optimal bandwidth was determined by minimizing the corrected AIC, as described by Fotheringham et al. [49].

GWR models produce a set of local regression results, including local intercepts, regression coefficients, residuals, R squared, and condition numbers, which can be mapped to show their spatial variability. GWR models were employed and mapped using ArcMap 10.

In a global model, whether regression coefficients significantly differ from zero is typically examined. This can be accomplished using a *t* test: the *t* statistics and their associated *p* values. A coefficient whose estimated value is found to be not significantly different from zero is associated with a variable whose variation does not contribute to the mode. Variables with nonsignificant regression coefficients can be eliminated from the model. The situation with GWR is slightly more complex. If one set of coefficients is associated with each regression point and with one set of standard errors, then potentially hundreds or thousands of tests would be required to determine whether coefficients are locally significant. The assumption behind the tests indicates that five in every hundred tests would be significant if a significance level of 0.05 is used [51].

The Benjamini-Hochberg (B-H) procedure for controlling the false discovery rate (FDR), which consistently modifies the significance level for each test, was suitably used to test the significance of local regression coefficients of GWR [51, 52]. Thissen et al. (2002) reported a rapid and easy method to calculate the FDR of the B-H procedure by using Microsoft Excel [52]. The B-H approach controls the FDR by sequentially comparing the observed *p* value for each family of multiple test statistics, in order from the largest to the smallest, to a list of computed B-H critical values [pB-H(i)]. The critical value on the list is determined for each test statistic, indexed by *i*, by linear interpolation between α/2 (for the largest observed *p* value) to (α/2)/m, where *m* is the family size (for the smallest of the *p* values). The last value is the Bonferroni critical value; thus, the reason for the gain in power of B-H relative to Bonferroni is clear. In the B-H approach, only the smallest of the *m* observed *p* values are compared with the Bonferroni critical value. All other *p* values are calculated using less stringent criteria [52]. The local regression coefficient is estimated to be significant if the *p* value is less than the B-H critical value; otherwise, it is deemed nonsignificant. The results of the significant determination of local regression coefficients were mapped using ArcMap 10.

### General statistics

We used Yate’s correction for chi-square tests [53] to analyze the variation in the incidence pattern (imported and autochthonous cases) among the 9 dengue outbreak years.

## Results

**Fig. 2:**
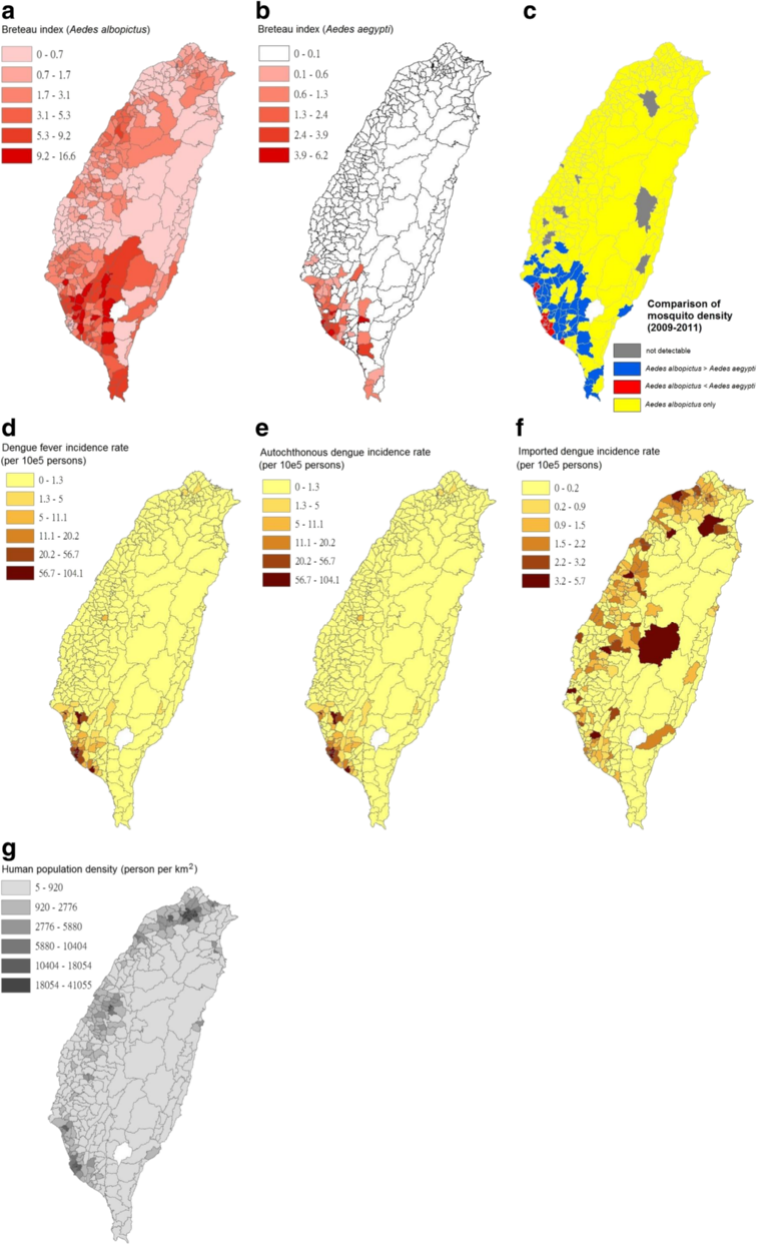
Maps of dengue fever incidence, *Aedes* mosquito density, and human population density in 348 townships in Taiwan during 2009–2011. **a** Breteau index (BI) of *Ae. albopictus* during 2009–2011. **b** BI of *Ae. aegypti* during 2009–2011. **c** Comparison between *Ae. albopictus* and *Ae. aegypti* density. **d** Incidence rates of all dengue fever (DF) cases during 2009–2011. **e** Incidence rates of autochthonous DF cases during 2009–2011. **f** Incidence rates of imported DF cases during 2009–2011. **g** Human population density during 2009–2011

Figure 2 presents a map showing the geographical distribution of the BIs of *Ae. albopictus* and *Ae. aegypti* per district; comparison of the density of *Ae. albopictus* and *Ae. aegypti*; incidence rates of DF, autochthonous dengue, and imported dengue cases; and density of human population between 2009 and 2011.

Table 1 lists the results of the global autocorrelation statistics for the variables of BI of *Ae. albopictus*; BI of *Ae. aegypti*; incidence rates of DF, autochthonous dengue, and imported dengue cases; and density of human population. The results of the global Moran’s test for all the variables were statistically significant, having a pseudo *p* value of less than 0.05 and indicating spatial heterogeneity (clustered).

**Table 1:** Global autocorrelation analysis of the studied variables in Taiwan during 2009–2011

Variable	Moran’s index	Z(I)	Pseudo *p* value	Description
*Aedes albopictus* (Breteau Index)	0.5718	17.46	0.0001	Clustered
*Aedes aegypti* (Breteau Index)	0.5818	18.03	0.0001	Clustered
dengue fever incidence rate	0.5587	17.75	0.0001	Clustered
autochthonous dengue incidence rate	0.5587	17.65	0.0001	Clustered
imported dengue incidence rate	0.1475	4.499	0.0002	Clustered
human population density	0.6623	20.54	0.0001	Clustered

Z(I) a value greater than 1.96 is considered statistically significant

**Fig. 3:**
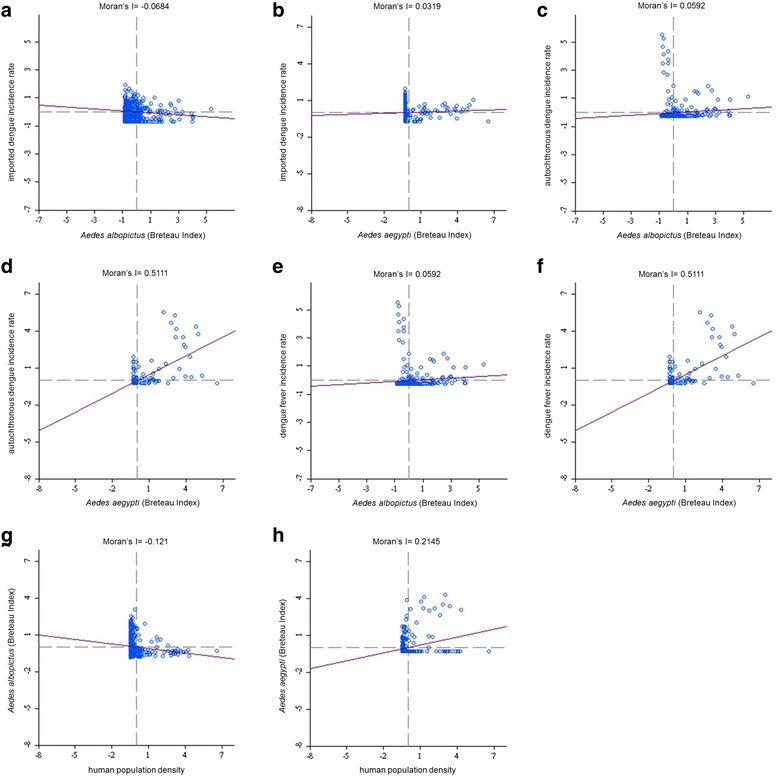
Moran’s I scatter plot calculated using the original variable and spatial lag as the second variable in Taiwan during 2009–2011. **a** Breteau index (BI) of *Ae. albopictus* and imported dengue incidence rate. **b** BI of *Ae. aegypti* and imported dengue incidence rate. **c** BI of *Ae. albopictus* and autochthonous dengue incidence rate. **d** BI of *Ae. aegypti* and autochthonous dengue incidence rate. **e** BI of *Ae. albopictus* and dengue fever incidence rate. **f** BI of *Ae. aegypti* and dengue fever incidence rate. **g** Human population density and BI of *Ae. albopictus*. **h** Human population density and BI of *Ae. aegypti*

According to the results of the global autocorrelation statistics, all the studied variables indicated spatial heterogeneity, which refers to the uneven distribution of a trait, event, or relationship across a region. The research methods of spatial dependence (e.g., global bivariate Moran’s I analysis) and spatial regression (e.g., GWR) were examined to investigate the relationship between the variables of *Aedes* mosquitoes and dengue incidences as well as between human population density and *Aedes* mosquitoes, which were implemented using eight types of combinations (Table 2). Global bivariate Moran’s I analysis was used to indicate variations in spatial distribution of data patterns and in cases where the correlation of two variables was investigated. The results of global bivariate Moran’s I analysis are listed in Table 2, with significance values based on a permutation approach and a corresponding *p* value of <0.05. Figure 3 depicts the corresponding global bivariate Moran’s I scatter plot. The slopes of the regression line in eight scatter plots equaled to Moran’s I indicator in Table 2 and differed from zero, indicating significant spatial correlation among the studied variables.

**Table 2:** Spatial dependence tests between the original variables and spatial lag as the second variables, which were calculated using global bivariate Moran’s I analysis in Taiwan during 2009–2011

Original variable	Spatial lag (second variable)	Bivariate Moran’s I	Pseudo *p*-value	Spatial dependence
*Aedes albopictus* (Breteau Index)	Imported dengue incidence rate	−0.07	0.0019	Negative
*Aedes aegypti* (Breteau Index)	Imported dengue incidence rate	0.03	0.0985	No correlation
*Aedes albopictus* (Breteau Index)	Autochthonous dengue incidence rate	0.06	0.0122	Positive
*Aedes aegypti* (Breteau Index)	Autochthonous dengue incidence rate	0.51	0.0001	Positive
*Aedes albopictus* (Breteau Index)	Dengue fever incidence rate	0.06	0.0139	Positive
*Aedes aegypti* (Breteau Index)	Dengue fever incidence rate	0.51	0.0001	Positive
Human population density	*Aedes albopictus* (Breteau Index)	−0.12	0.0001	Negative
Human population density	*Aedes aegypti* (Breteau Index)	0.21	0.0001	Positive

**Fig. 4:**
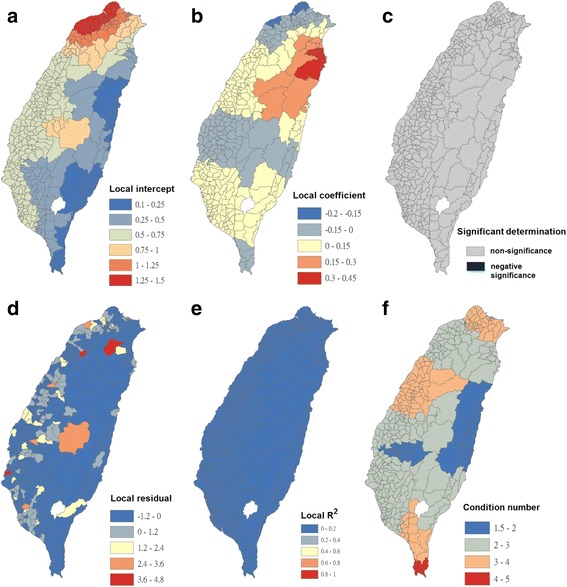
Results of the GWR model for Breteau indices of *Ae. albopictus* (as the explanatory variable) and imported incidence of dengue fever (as the response variable) in 348 townships in Taiwan during 2009–2011. **a** Local intercepts during 2009–2011. **b** Local regression coefficients during 2009–2011. **c** Significant determination of coefficients according to the Benjamini–Hochberg false discovery rate during 2009–2011. **d** Local residuals during 2009–2011. **e** Local R^2^ values during 2009–2011. **f** Local condition number values during 2009–2011

**Fig. 5:**
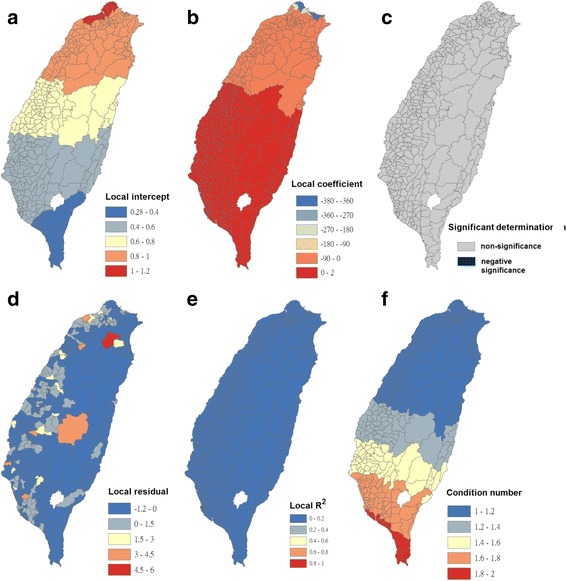
Results of the GWR model for Breteau indices of *Ae. aegypti* (as the explanatory variable) and the imported incidence of dengue fever (as the response variable) in 348 townships in Taiwan during 2009–2011. **a** Local intercepts during 2009–2011. **a** Local regression coefficients during 2009–2011. **c** Significant determination of coefficients according to the Benjamini-Hochberg false discovery rate during 2009–2011. **d** Local residuals during 2009–2011. **e** Local R^2^ values during 2009–2011. **f** Local condition number values during 2009–2011

**Fig. 6:**
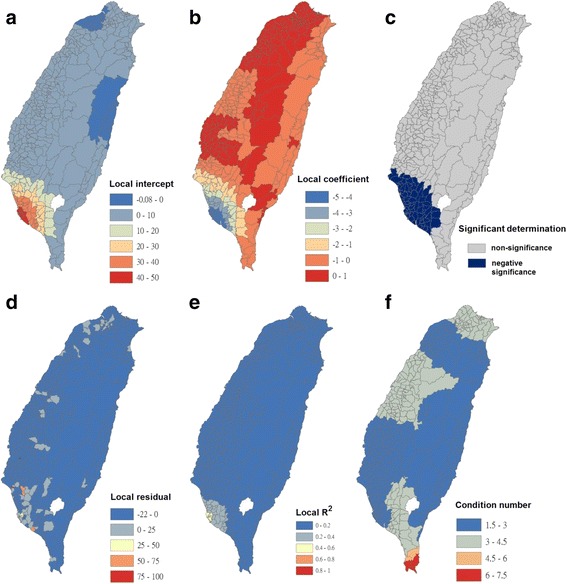
Results of the GWR model for Breteau indices of *Ae. albopictus* (as the explanatory variable) and autochthonous incidence of dengue fever (as the response variable) in 348 townships in Taiwan during 2009–2011. **a** Local intercepts during 2009–2011. **b** Local regression coefficients during 2009–2011. **c** Significant determination of coefficients according to the Benjamini–Hochberg false discovery rate during 2009–2011. **d** Local residuals during 2009–2011. **e** Local R^2^ values during 2009–2011. **f** Local condition number values during 2009–2011

**Fig. 7:**
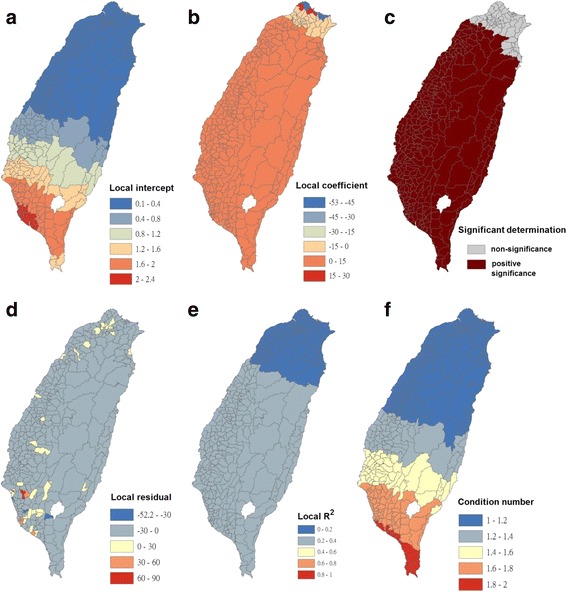
Results of the GWR model for Breteau indices of *Ae. aegypti* (as the explanatory variable) and autochthonous incidence of dengue fever (as the response variable) in 348 townships in Taiwan during 2009–2011. **a** Local intercepts during 2009–2011. **b** Local regression coefficients during 2009–2011. **c** Significant determination of coefficients according to the Benjamini–Hochberg false discovery rate during 2009–2011. **d** Local residuals during 2009–2011. **e** Local R^2^ values during 2009–2011. **f** Local condition number values during 2009–2011

**Fig. 8:**
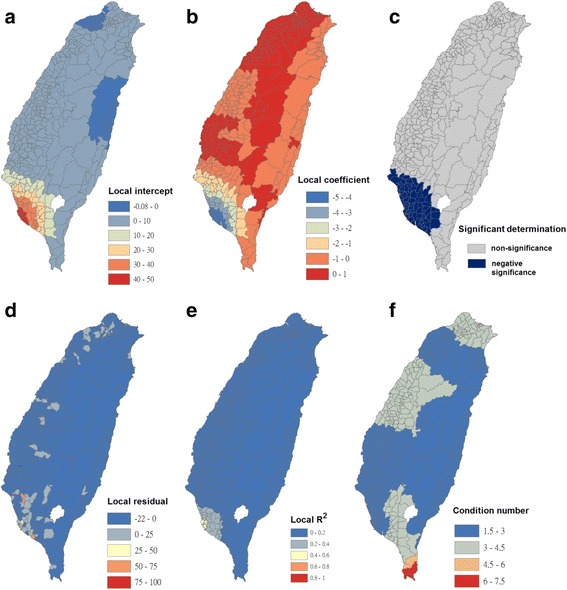
Results of the GWR model for Breteau indices of *Ae. albopictus* (as the explanatory variable) and dengue fever incidence rates (as the response variable) in 348 townships in Taiwan during 2009–2011. **a** Local intercepts during 2009–2011. **b** Local regression coefficients during 2009–2011. **c** Significant determination of coefficients according to the Benjamini–Hochberg false discovery rate during 2009–2011. **d** Local residuals during 2009–2011. **e** Local R^2^ values during 2009–2011. **f** Local condition number values during 2009–2011

**Fig. 9:**
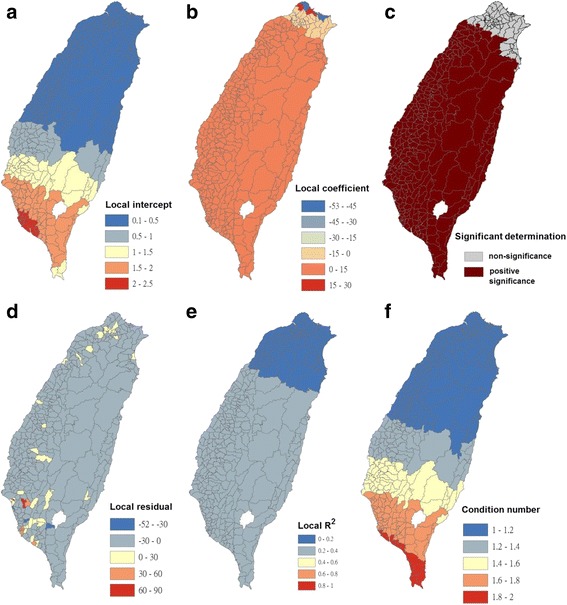
Results of the GWR model for Breteau indices of *Ae. aegypti* (as the explanatory variable) and dengue fever incidence rates (as the response variable) in 348 townships in Taiwan during 2009–2011. **a** Local intercepts during 2009–2011. **b** Local regression coefficients during 2009–2011. **c** Significant determination of coefficients according to the Benjamini-Hochberg false discovery rate during 2009–2011. **d** Local residuals during 2009–2011. **e** Local R^2^ values during 2009–2011. **f** Local condition number values during 2009–2011

**Fig. 10:**
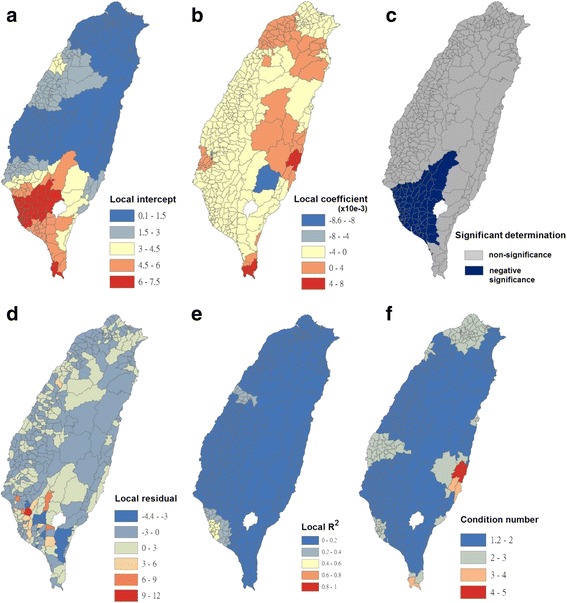
Results of the GWR model for human population densities (as the explanatory variable) and Breteau indices of *Ae. albopictus* (as the response variable) in 348 townships in Taiwan during 2009–2011. **a** Local intercepts during 2009–2011. **b** Local regression coefficients during 2009–2011. **c** Significant determination of coefficients according to the Benjamini-Hochberg false discovery rate during 2009–2011. **d** Local residuals during 2009–2011. **e** Local R^2^ values during 2009–2011. **f** Local condition number values during 2009–2011

**Fig. 11:**
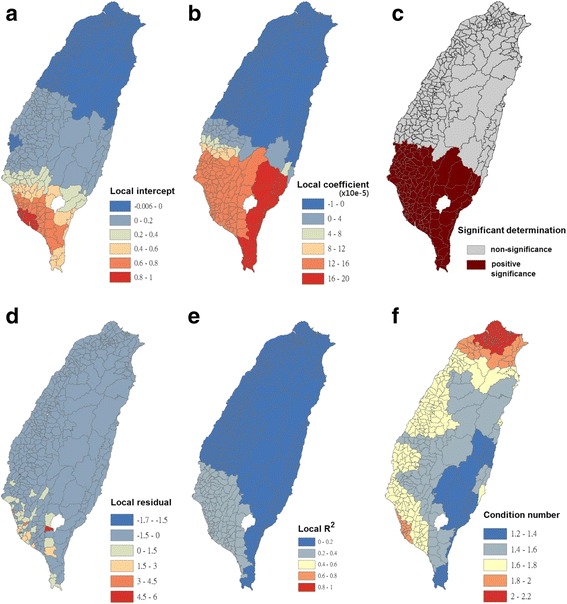
Results of the GWR model for human population densities (as the explanatory variable) and Breteau indices of *Ae. aegypti* (as the response variable) in 348 townships in Taiwan during 2009–2011. **a** Local intercepts during 2009–2011. **b** Local regression coefficients during 2009–2011. **c** Significant determination of coefficients according to the Benjamini–Hochberg false discovery rate during 2009–2011. **d** Local residuals during 2009–2011. **e** Local R^2^ values during 2009–2011. **f** Local condition number values during 2009–2011

Condition numbers indicate when results are unstable because of local multicollinearity and the sensitivity of a linear equation solution to small changes in matrix coefficients. The condition number included in the GWR output indicates when local collinearity is a problem. A condition number of >30 is not reliable. In this study, condition numbers were calculated using the GWR analysis, as presented in Figs. 4f, 5f, 6f, 7f, 8f, 9f, 10f and 11f. The values ranged from 1 to 7.5. All the outcomes of fitting the GWR models were reliable.

The maps in Fig. 4 are presented as local intercepts, local regression coefficients, significant B-H FDR values, local residuals, local *R* ^2^ values, and condition number values, in which the imported incidence of DF fits the GWR models with the explanatory variable of the BI of *Ae. albopictus* during 2009–2011. As presented in Fig. 4, regression coefficients of the incidence rate of *Ae. albopictus* were not significant in all study areas (local *R* ^2^ < 0.09). The results indicated that local *Ae. albopictus* did not contribute to imported dengue cases in the main island of Taiwan. We used bivariate Moran’s I analysis to examine spatial dependence between the imported incidence of DF and BI of *Ae. albopictus*, and the results were negatively significant and demonstrated low correlation (bivariate Moran’s index = −0.07; Table 2 and Fig. 3a).

The maps in Fig. 5 are presented as local intercepts, local regression coefficients, significant B-H FDR values, local residuals, local *R* ^2^ values, and condition number values, in which the imported incidence of DF fits the GWR models with the explanatory variable of the BI of *Ae. aegypti* during 2009–2011. As presented in Fig. 5, regression coefficients of the incidence rate of *Ae. aegypti* were not significant in all study areas (local *R* ^2^ < 0.08). The results indicated that local *Ae. aegypti* did not contribute to imported dengue cases in the main island of Taiwan. We used bivariate Moran’s I analysis to examine spatial dependence between the imported incidence of DF and BI of *Ae. aegypti*, and the results exhibited no correlation (bivariate Moran’s index = 0.03; Table 2 and Fig. 3b).

The maps in Fig. 6 are presented as local intercepts, local regression coefficients, significant B-H FDR values, local residuals, local *R* ^2^ values, and condition number values, in which the autochthonous incidence of DF fits the GWR models with the explanatory variable of the BIs of *Ae. albopictus* during 2009–2011. As presented in Fig. 6, regression coefficients of the incidence rate of *Ae. albopictus* were negatively significant, with clusters covering most areas in the western area of southern Taiwan (local *R* ^2^ < 0.45). The results indicated that *Ae. albopictus* did not positively contribute to autochthonous dengue cases on the main island of Taiwan. We used bivariate Moran’s I analysis to examine spatial dependence between the autochthonous incidence of DF and BI of *Ae. albopictus*, and the results were positively significant but exhibited low correlation (bivariate Moran’s index = 0.06; Table 2 and Fig. 3c).

The maps in Fig. 7 are presented as local intercepts, local regression coefficients, significant B-H FDR values, local residuals, local *R* ^2^ values, and condition number values, in which the autochthonous incidence of DF fits the GWR models with the explanatory variable of the BIs of *Ae. aegypti* during 2009–2011. As presented in Fig. 7, regression coefficients of the incidence rate of *Ae. aegypti* were positively significant, with clusters covering most areas of Taiwan. A total of 290 townships correlated with local autochthonous dengue incidence (local *R* ^2^ < 0.35). The results indicated that autochthonous DF in Taiwan was transmitted by *Ae. aegypti*. Furthermore, the results of bivariate Moran’s I analysis revealed that spatial dependence between the autochthonous incidence of DF and BI of *Ae. aegypti* was positively significant (bivariate Moran’s index = 0.51; Table 2 and Fig. 3d).

The maps in Fig. 8 are presented as local intercepts, local regression coefficients, significant B-H FDR values, local residuals, local *R* ^2^ values, and condition number values, in which the DF incidence fits the GWR models with the explanatory variable of the BI of *Ae. albopictus* during 2009–2011. As presented in Fig. 8, regression coefficients of the incidence rate of *Ae. albopictus* were negatively significant, with clusters covering most areas in the western area of southern Taiwan (local R^2^ < 0.45)*.* The results indicated that *Ae. albopictus* did not positively contribute to dengue cases in the main island of Taiwan. We used bivariate Moran’s I analysis to examine spatial dependence between the incidence of DF and BI of *Ae. albopictus*, and the results were positively significant but had very low correlation (bivariate Moran’s index = 0.06; Table 2 and Fig. 3e).

The maps in Fig. 9 are presented as local intercepts, local regression coefficients, significant B-H FDR values, local residuals, local *R* ^2^ values, and condition number values, in which the incidence of DF fits the GWR models with the explanatory variable of the BI of *Ae. aegypti* during 2009–2011. As illustrated in Fig. 9, regression coefficients of the incidence rate of *Ae. aegypti* were positively significant, with clusters covering most areas of Taiwan. A total of 290 townships were correlated with DF incidence (local *R* ^2^ < 0.35). The results indicated that DF incidence in Taiwan was transmitted by *Ae. aegypti*. We used bivariate Moran’s I analysis to examine spatial dependence between the incidence of DF and BI of *Ae. aegypti*, and the results were positively significant (bivariate Moran’s index = 0.51; Table 2 and Fig. 3f).

The maps in Fig. 10 are presented as local intercepts, local regression coefficients, significant B-H FDR values, local residuals, local *R* ^2^ values, and condition number values, in which the BI of *Ae. albopictus* fit the GWR models with the explanatory variable of human population density during 2009–2011. As presented in Fig. 10, regression coefficients of the human population density were negatively significant, with clusters covering most areas of plain and mountainous townships in the south part of Taiwan (local R^2^ < 0.5). The results indicated that a high density of human population did not positively contribute to the prevalence of *Ae. albopictus*; however, it reduced the mosquito density in the south part of Taiwan. We used bivariate Moran’s I analysis to examine spatial dependence between the BI of *Ae. albopictus* and density of human population, and the results were negatively significant (bivariate Moran’s index = −0.12; Table 2 and Fig. 3g).

The maps in Fig. 11 are presented as local intercepts, local regression coefficients, significant B-H FDR values, local residuals, local *R* ^2^ values, and condition number values, in which the BI of *Ae. aegypti* fits the GWR models with the explanatory variable of the human population density during 2009–2011. As illustrated in Fig. 11, regression coefficients of the human population density were positively significant, with clusters covering most areas of Taiwan’s townships in the south of the Tropic of Cancer (i.e., 23.5°N; local R^2^ < 0.33). The results indicated that the human population density contributed to the incidence of *Ae. aegypti* in southern Taiwan. We used bivariate Moran’s I analysis to examine spatial dependence between the BI of *Ae. aegypti* and density of human population, and the results were positively significant (bivariate Moran’s index = 0.21; Table 2 and Fig. 3h).

We used Yates’ chi-square test to examine the patterns of confirmed dengue cases (imported or autochthonous) among 9 dengue outbreak years (i.e., 2002, 2006, 2007, 2009, 2010, 2011, 2012, 2014, and 2015); dengue outbreak years are defined as those having >1000 confirmed dengue cases. The two-sided test had a significance level of 0.05. The results indicated that most of the years had dissimilar incidence patterns. However, few tests did not reject the null hypothesis, and the years with similar incidence patterns were classified into three groups: “2006, 2007, and 2011”; “2010 and 2012”; and “2002 and 2015,” as shown in Table 3.

**Table 3:** Yates’ chi-square test and matric comparisons of nine dengue outbreak years^a^

Year	2002	2006	2007	2009	2010	2011	2012	2014	Year	Imported case (Im)	autochthonous case (Au)
	(Im/Au)	(Im/Au)	(Im/Au)	(Im/Au)	(Im/Au)	(Im/Au)	(Im/Au)	(Im/Au)	2002	52	5336
2006 (Im/Au)	*307.13								2006	109	965
2007 (Im/Au)	*273.09	3.1							2007	179	2000
2009 (Im/Au)	*778.08	*35.43	*83.76						2009	204	848
2010 (Im/Au)	*681.82	*19.34	*58.58	*5.12					2010	304	1592
2011 (Im/Au)	*305.53	0.55	1.11	*58.12	*36.62				2011	157	1545
2012 (Im/Au)	*539.76	*8.17	*30.67	*12.71	2.5	*17.37			2012	207	1271
2014 (Im/Au)	*8.84	*363.42	*371.93	*1215.21	*1186.02	*405.63	*335.02		2014	240	15492
2015 (Im/Au)	0.84	*861.18	*960.62	*2808.97	*2899.05	*1009.39	*1242.15	*54.38	2015	365	43419

*Im* imported case, *Au* autochthonous case

Significance (*) is a chi-square value > 3.84 (*p* value = 0.05)

^a^indicates the number of confirmed dengue > 1000 cases

Data extracted from Taiwan CDC [37]

## Discussion

Extensive and rapid urbanization without appropriate planning may have directly resulted in large numbers of artificial containers that are suitable for breeding *Aedes* mosquitoes around households. The rapidly developing air transportation has accelerated virus translocation, enabling it to easily migrate from endemic to non-endemic areas [54]. Therefore, the probability of virus introduction through imported cases has considerably increased, even in countries where dengue has never been reported. Moreover, travelers infected with DENV serve as vehicles for its potential transmission [55]. Other environmental factors, such as global warming, have been associated with the occurrence of dengue epidemics [56, 57], causing this disease to be more complicated and thus more difficult to manage. Socioeconomic factors affecting the distribution of *Aedes* mosquitoes, other than the use of containers to store water, include air conditioner use, housing quality, and urbanization rate [58, 59]. *Ae. aegypti* and *Ae. albopictus* are found in both urban and rural areas. *Ae. aegypti* tends to breed in urbanized ecological niches, whereas *Ae. albopictus* is more frequently found in rural areas; however, mixed breeding has been reported [60]. In addition, *Ae. aegypti* prefers living indoors, whereas *Ae. albopictus* frequently breeds outdoors [61–63]. Dengue outbreaks have occurred nearly every year in Taiwan since 1987, first appearing in the southern part of Taiwan, which has a high human population density. Dengue outbreaks usually increase during rapid urbanization, and a high human population density has been associated with dengue transmission because of its potential impact on human-mosquito contact [64, 65]. Our study results revealed that the clusters of *Ae. aegypti* were associated with a high density of human population in the southern part of Taiwan, where the risk of dengue outbreaks is high (Figs. 2, 10, and 11). *Ae. aegypti* has more opportunities to increase human-mosquito contact and thus increase the probability of dengue outbreaks. However, the opposite results were observed for *Ae. albopictus*.

Studies on the spatial coexistence of *Ae. aegypti* and *Ae. albopictus* have reported that the two species are sympatric [66, 67]. In North America [68] and Brazil [66], the two species have similar larval ecological niches and often share the same larval habitat. Likewise, in Mayotte, *Ae. albopictus* coexists with *Ae. aegypti* in 40 % of larval habitats [69]. However, as suggested by Paupy et al. [70], the apparent coexistence of the two species could be a transient situation, followed by a reduction [71–73] or displacement [74, 75] of the resident species; interspecific larval competition for resources is the most likely reason for this process. Moreover, a few local studies have reported that the local spread of *Ae. albopictus* and decline in *Ae. aegypti* populations might be linked to interspecies competition [68, 76, 77] or non-reciprocal cross-species inseminations [78].

*Ae. aegypti* almost exclusively feeds on humans in daylight hours and typically rests indoors [79]. By contrast, *Ae. albopictus* is usually exophagic and bites humans and animals opportunistically [70]; however, *Ae. albopictus* has been reported to exhibit strongly anthropophilic behavior similar to that of *Ae. aegypti* in specific contexts [63, 80]. Both species inhabit containers but differ in their behavior and biology; thus, they occupy different niches [81]. Studies have reported that compared with *Ae. aegypti*, which is usually endophagic, *Ae. albopictus* tends to be exophagic and thus prefers to feed on blood indoors [62, 80]. Multiple blood feedings occur frequently in *Ae. albopictus* and *Ae. Aegypti*, which usually becomes engorged after two or three blood meals [80, 82–84]. However, multiple blood feedings in a single gonotrophic cycle are more frequent in *Ae. aegypti* [63, 82, 85]. Such behavioral differences may provide fewer opportunities for *Ae. albopictus* to contact humans infected by DENV. Thus, *Ae. albopictus* has a lower probability of transmitting the virus through the intake of a blood meal [86]. Mosquito behaviors are crucial in determining the occurrence and scale of many arthropod-borne diseases and diseases caused by nematodes, protozoans, and viruses [87].

Both *Ae. albopictus* and *Ae. aegypti* are susceptible to DENV. However, rates of salivary gland infection and transmission are higher in *Ae. aegypti*-fed blood meals than in *Ae. albopictus*-fed blood meals containing DENV, either in mosquitoes from Taiwan or Southeast Asian countries [86, 88]. A study on field-caught mosquitoes conducted in Singapore reported that 6.9 % of *Ae. aegypti*, but only 2.9 % of *Ae. albopictus* was positive for DENVs [89]. Another study conducted in Singapore reported that infected *Ae. aegypti* were detected as early as six weeks before the start of a dengue outbreak, whereas infected *Ae. albopictus* did not appear until the number of cases was increasing [21]. Furthermore, a study conducted in southern Taiwan demonstrated that DENV was detected in low levels only from 0.2 % positive signs for field-caught *Ae. aegypti*, but not for *Ae. albopictus* [90]. Large outbreaks such as those in 2006 and 2007 occurred one year after the detection of virus-infected *Ae. aegypti* [90]. These results prompt the possibility that *Ae. aegypti* can efficiently establish a preliminary dengue case cluster. Because of frequent subclinical or cryptic infections, dengue outbreaks may be delayed or not reported, resulting in silent transmission [91]. In addition, competition for resources by other intracellular organisms is crucial for mosquitoes to support viral replication [92]. In Taiwan, the endosymbiont Wolbachia has been identified in nearly all *Ae. albopictus* populations; however, it is completely absent from *Ae. aegypti* populations breeding in the field [93]. The artificial introduction of an exogenous strain of Wolbachia, leading to competition for cellular resources, interfered with replication of DENV in *Ae. aegypti*. Wolbachia-mediated pathogen interference may synergistically work with the life-shortening strategy [92]. This suggests that this maternally inherited endosymbiont acts as a factor determining viral infection and its dissemination to other tissues; that is, from the midgut to the salivary glands of the mosquito [94, 95]. In addition, the study results in Malaysia revealed transovarial transmission of dengue virus in the larvae of *Ae. aegypti* and *Ae. albopictus* in the field, with 6.3 % positive results [96]. However, according to a study of virological surveillance in Taiwan, transovarial transmission in local *Aedes* mosquitos may not occur, or occur at lower rates [90].

Since the epidemic in 1987, few small outbreaks have been reported in north of the Tropic of Cancer, such as those in Chungho (i.e., Jhonghe City) in Taipei County in 1995 (162 cases), Tunghai University in Taichung City in 1995 (8 cases), and Taipei City in 1996 (14 cases); these outbreaks occurred in areas without *Ae. aegypti* [97, 98]. Because *Ae. albopictus* is also susceptible to DENV [86, 99], it may also cause sporadic cases, and occasionally, small outbreaks. In turn, only a few autochthonous cases can occur and thus the scales of outbreaks are limited in northern Taiwan, although more imported cases are reported every year in that area. Despite similarities between *Ae. aegypti* and *Ae. albopictus*, they differ in crucial characteristics, including behaviors and vector competence to the dengue virus. Such differences may cause discrepancies in their roles in dengue outbreaks. Considering the occurrence of DF in past decades in Taiwan, only limited or no outbreaks can be established in which *Ae. aegypti* is absent (northern and central parts of Taiwan where *Ae. albopictus* is prevalent). Yang et al. (2014) [100] hypothesized that the existence of *Ae. aegypti* is a prerequisite for the initiation and establishment of a dengue outbreak, which may be expanded or maintained through participation of *Ae. albopictus*. The outbreak may be terminated when herd immunity in the resident population reaches a level sufficiently high to block the spread of infections. However, this hypothesis is inconsistent with our results presented in Figs. 6 and 7. The clusters in dengue-endemic areas of southern Taiwan were located where the incidence of autochthonous cases was negatively correlated with *Ae. albopictus* and positively correlated with *Ae. aegypti*. Our findings revealed that *Ae. aegypti* in endemic areas played a critical role in establishing most scales of dengue outbreaks during 2009–2011. However, *Ae. albopictus* may have a supporting role or cause sporadic cases and occasionally small outbreaks in the absence of *Ae. aegypti*.

## Conclusions

*Ae. aegypti* is a key vector for dengue epidemics in Taiwan. According to the BIs of *Ae. aegypti*, but not of *Ae. albopictus*, high-density clusters of human populations are highly associated with the emergence of dengue epidemics in southern Taiwan. *Ae. albopictus* may have a supporting role during small-scale dengue outbreaks or cause sporadic cases and occasionally small outbreaks in the absence of *Ae. aegypti*. This observation may provide broader considerations for health authorities responsible for dengue prevention, hopefully leading to a more efficient allocation of resources, particularly in the control of mosquito vectors.
